# Predicting invasive fungal disease due to *Candida* species in non-neutropenic, critically ill, adult patients in United Kingdom critical care units

**DOI:** 10.1186/s12879-016-1803-9

**Published:** 2016-09-09

**Authors:** Jason Shahin, Elizabeth J. Allen, Krishna Patel, Hannah Muskett, Sheila E. Harvey, Jonathan Edgeworth, Christopher C. Kibbler, Rosemary A. Barnes, Sharmistha Biswas, Neil Soni, Kathryn M. Rowan, David A. Harrison, FIRE Study Investigators

**Affiliations:** 1 Intensive Care National Audit & Research Centre, Napier House, 24 High Holborn, London, WC1V 6AZ UK; 2 McGill University, Montreal, Canada; 3 Department of Medical Statistics, London School of Hygiene and Tropical Medicine, London, UK; 4 St Thomas’ Hospital, London, UK; 5 Royal Free Hospital, London, UK; 6 Cardiff University, Cardiff, UK; 7 Chelsea and Westminster Hospital, London, UK

**Keywords:** Critical care, Invasive fungal disease, Candida, Fungal, Prophylaxis

## Abstract

**Background:**

Given the predominance of invasive fungal disease (IFD) amongst the non-immunocompromised adult critically ill population, the potential benefit of antifungal prophylaxis and the lack of generalisable tools to identify high risk patients, the aim of the current study was to describe the epidemiology of IFD in UK critical care units, and to develop and validate a clinical risk prediction tool to identify non-neutropenic, critically ill adult patients at high risk of IFD who would benefit from antifungal prophylaxis.

**Methods:**

Data on risk factors for, and outcomes from, IFD were collected for consecutive admissions to adult, general critical care units in the UK participating in the Fungal Infection Risk Evaluation (FIRE) Study. Three risk prediction models were developed to model the risk of subsequent *Candida* IFD based on information available at three time points: admission to the critical care unit, at the end of 24 h and at the end of calendar day 3 of the critical care unit stay. The final model at each time point was evaluated in the three external validation samples.

**Results:**

Between July 2009 and April 2011, 60,778 admissions from 96 critical care units were recruited. In total, 359 admissions (0.6 %) were admitted with, or developed, *Candida* IFD (66 % *Candida albicans*). At the rate of candidaemia of 3.3 per 1000 admissions, blood was the most common *Candida* IFD infection site. Of the initial 46 potential variables, the final admission model and the 24-h model both contained seven variables while the end of calendar day 3 model contained five variables. The end of calendar day 3 model performed the best with a c index of 0.709 in the full validation sample.

**Conclusions:**

Incidence of Candida IFD in UK critical care units in this study was consistent with reports from other European epidemiological studies, but lower than that suggested by previous hospital-wide surveillance in the UK during the 1990s. Risk modeling using classical statistical methods produced relatively simple risk models, and associated clinical decision rules, that provided acceptable discrimination for identifying patients at ‘high risk’ of Candida IFD.

**Trial registration:**

The FIRE Study was reviewed and approved by the Bolton NHS Research Ethics Committee (reference: 08/H1009/85), the Scotland A Research Ethics Committee (reference: 09/MRE00/76) and the National Information Governance Board (approval number: PIAG 2-10(f)/2005).

**Electronic supplementary material:**

The online version of this article (doi:10.1186/s12879-016-1803-9) contains supplementary material, which is available to authorized users.

## Background

Once seen typically in immunocompromised patients, invasive fungal disease (IFD) amongst the critically ill is now predominantly found in the adult, general, non-immunocompromised population [1]. Over 5000 cases of IFD due to *Candida* species (*Candida* IFD) occur in the UK each year, with 40 % occurring in critical care units [2]. Epidemiological trends in IFD among the critically ill are changing, due in large part to the increased incidence of IFD risk factors and to new therapeutic strategies.

A number of randomised controlled trials have evaluated prophylactic and or empiric therapy with antifungals in non-neutropenic, critically ill patients. Despite patient heterogeneity, these trials have demonstrated a beneficial effect of antifungal prophylaxis on the risk of developing proven IFD and suggested a reduction in mortality [3]. Given that the effectiveness of antifungal prophylaxis has only been demonstrated in groups at high risk of IFD and that more widespread use of antifungal drugs may promote resistance and drive up costs, it is necessary to establish a method to identify high risk patient groups, who stand to benefit most from an antifungal prophylactic strategy. Several risk models and clinical decision rules have been proposed for identifying patients at high risk of IFD [4–8]. However, model generalisability has been limited owing to the restricted study population, being either post-surgical patients [7, 8], or patients already colonised with *Candida* [8].

Given the potential benefit of antifungal prophylaxis and lack of generalisable tools to identify high risk patients, the aim of the current study was to describe the epidemiology of IFD in UK critical care units and to develop and validate a clinical risk prediction tool to predict the risk of *Candida* IFD at three decision time points: at admission to the critical care unit, at 24 h following admission, and at the end of the third calendar day following admission, in order to identify non-neutropenic, critically ill adult patients at high risk of IFD who would benefit from antifungal prophylaxis.

## Methods

### Study design

Based on the results of a systematic review of the literature conducted by our research team [5] and on expert clinical opinion, data on risk factors for, and outcomes from, IFD were collected for consecutive admissions to adult, general critical care units in the UK participating in the Fungal Infection Risk Evaluation (FIRE) Study [9]. These data were linked with additional patient data collected for national clinical audits – the Case Mix Programme (in England, Wales and Northern Ireland) and the Scottish Intensive Care Society Audit Group (in Scotland) – to form the FIRE Study database. For all sources, data were collected prospectively and abstracted by trained data collectors and underwent extensive validation.

### FIRE Study database

For all critical care unit admissions, data were extracted on age, sex, medical history, surgical status, acute severity of illness, primary reason for admission, therapies received, IFD, fungal colonisation, mortality and length of stay.

Severe comorbidities were defined using the APACHE II method [10] and must have been evident in the 6 months prior to critical care unit admission. Surgery within up to 7 days prior to admission to the critical care unit was classified as either emergency/urgent or scheduled/elective. Acute severity of illness was summarised using the APACHE II Score [10] and the ICNARC Physiology Score [11] assessed during the first 24 h following admission to the critical care unit.

Data on the following therapies were collected: total parenteral nutrition; systemic antimicrobials; immunosuppressive therapy; central venous catheters, organ support; and antifungal use. Corticosteroids were included as immunosuppressives. Organ support was recorded throughout the critical care unit stay, defined according to the UK Department of Health Critical Care Minimum Dataset [12].

Fungal colonisation was defined as the presence of yeasts in any sample reported on a microbiology system and was recorded as the date that a positive report was available – i.e. the point at which a treatment decision could be made based on this knowledge.

IFD was defined as a blood culture or sample from a normally sterile site (including, but not restricted to: cerebrospinal fluid; peritoneal fluid; pleural fluid; and pericardial fluid; and excluding bronchoalveolar lavage, urine and sputum) positive for yeast/mould cells in a microbiological or histopathological report. This definition was based on the Revised Definitions of Invasive Fungal Disease from the European Organization for Research and Treatment of Cancer/Invasive Fungal Infections Cooperative Group and the National Institute of Allergy and Infectious Diseases Mycoses Study Group (EORTC/MSG) Consensus Group [13]. This definition was chosen to best capture *Candida* IFD and was recognised to under-represent IFD due to other species. Timing of IFD was defined by the date on which the positive sample was collected. All patients reported to have IFD (pre critical care unit or during critical care unit stay), and a random sample of 2 % of those reported not to have IFD, were independently rechecked against hospital notes and microbiology records by the local investigator, blinded to the original data. For admissions that developed IFD during critical care unit stay, the timing of the first systemic antifungal was also reported relative to the timing of IFD.

Patients were followed up for mortality and length of stay until death or final discharge from acute hospital and those transferred to another acute hospital.

### Descriptive epidemiology

Analyses were performed using Stata/SE Version 10.1 (StataCorp LP, College Station TX). For the purpose of summarising case mix, outcomes and antifungal use, the cohort was divided into groups of: admissions with IFD positive for *Candida albicans* (*Candida albicans* IFD); admissions with IFD positive for other *Candida* species (non-albicans *Candida* spp IFD); and admissions with no IFD either prior to or during the critical care unit stay (no IFD). Admissions with IFD positive for *Candida* of unknown species or non-*Candida* species were excluded due to small numbers. Admissions with IFD positive for both *Candida albicans* and non-albicans *Candida* species were included in the *Candida albicans* subgroup.

### Development and validation of risk prediction models

Three risk prediction models were developed to model the risk of subsequent *Candida* IFD based on information available at three time points: admission to critical care unit, at the end of 24 h and at the end of calendar day 3 of the critical care unit stay.

The following exclusions were applied for the development and validation of the risk model at admission: age less than 18 years; second and subsequent admissions of the same patient; neutropenia (absolute neutrophil count less than 1 × 10^9^ l^−1^); active haematological malignancy; admission following solid organ transplant; IFD identified up to 7 days prior to admission; and receipt of systemic antifungals up to 7 days prior to admission.

For the models at 24 h and at the end of calendar day 3, exclusions were as above plus any of the following occurring before the decision time point: death or discharge from the critical care unit; IFD; or receipt of systemic antifungals.

The dataset was divided into the following development and validation samples: development sample – all admissions to a random sample of participating critical care units in England, Wales and Northern Ireland, July 2009 to December 2010 (selected to include approximately two thirds of all admissions); random validation sample – all admissions to the remaining units in England, Wales and Northern Ireland; temporal validation sample – all admissions to units in the development sample, January to March 2011; and geographical validation sample – all admissions to units in Scotland.

The risk prediction models were derived in the development sample using logistic regression models with robust standard errors to allow for clustering within critical care units. All candidate variables were included in a ‘full’ multivariable model and the model was progressively simplified using backwards-stepwise selection with the least statistically significant being removed at each step.

Model discrimination was assessed with the c index [14], equivalent to the area under the receiver operating characteristic curve [15], calibration by graphical plots of observed against expected risk, and overall fit by Brier’s score [16], the mean squared error between outcome and prediction. Bootstrapping with 200 bootstrap samples was used on the development sample to internally validate the final selected model at each time point and to estimate optimism adjusted measures of the model discrimination and overall fit [17]. The final model at each time point was evaluated in the three external validation samples: a random sample, a geographic sample and a temporal sample, as described above. Each sample was chosen to test different aspects of future performance. The models were evaluated in each validation sample separately and then in all three samples combined using the same measures as in the development sample.

The sensitivity, specificity, positive predictive value and negative predictive value of the risk model at the end of calendar day 3 was compared with that of existing clinical decision rules identified from the systematic review of the literature using the full validation dataset. For comparison with the clinical decision rules, three alternative risk thresholds were applied to the risk predictions from the FIRE Study model corresponding to predicted risks of >0.5 % (F1), >1 % (F2) and >2 % (F3). The following existing clinical decision rules were included in the comparison: three rules presented in Ostrosky-Zeichner et al. [6] (OZ1-OZ3) and three rules presented in Paphitou et al. [7] (P1-P3).

## Results

**Fig. 1:**
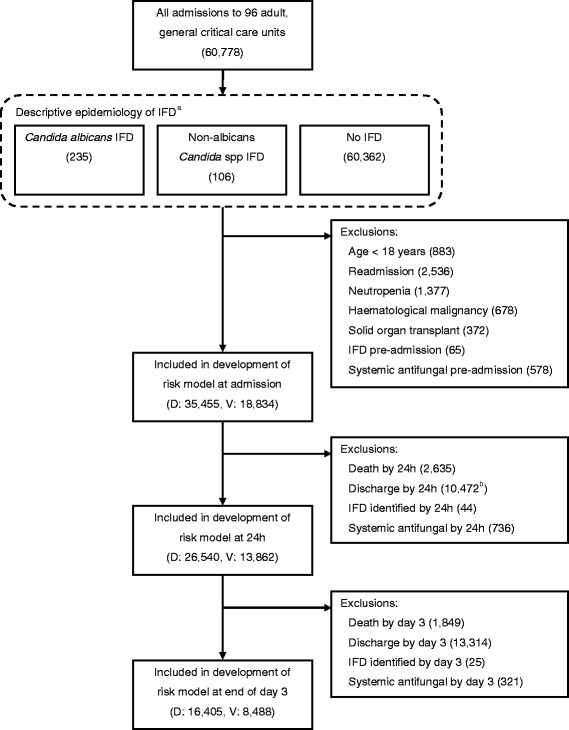
Flow of admissions in the FIRE Study

Between July 2009 and April 2011, a total of 96 critical care units with 60,778 admissions participated in the FIRE Study (see Fig. 1). The units were representative of all UK adult general critical care units in terms of geographical distribution, hospital teaching status and number of beds.

**Fig. 2:**
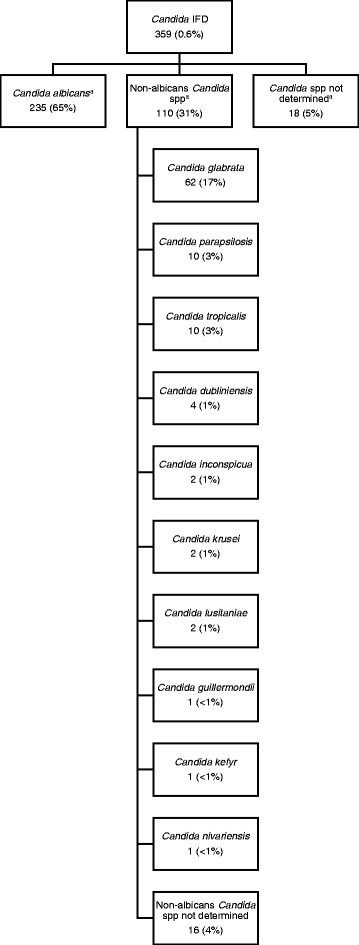
Organogram of *Candida* species causing invasive fungal disease

In total, 359 admissions (0.6 %) were admitted with, or developed, *Candida* IFD of which 66 % were *Candida albicans* (see Fig. 2). The most common non-albicans *Candida* species was *Candida glabrata* (17 %).

Admissions with non-albicans *Candida* spp IFD were compared to admissions with *Candida albicans* IFD as shown in Table 1. The critical care unit and acute hospital lengths of stay and mortality rates were comparable between the *Candida albicans* and non-albicans *Candida* spp. IFD subgroups but substantially higher when compared with admissions with no IFD. Overall crude critical care unit and acute hospital mortality for admissions with any *Candida* species IFD were 29.9 and 39.6 %, respectively.

**Table 1:** Case mix of admissions by invasive fungal disease subgroup

	*Candida albicans* IFD	Non-albicans *Candida* spp. IFD	No IFD
Number of admissions	235	106	60,362
Demographics			
Age (years), median (IQR)	61 (49, 71)	62 (50, 73)	64 (48, 74)
Male sex, n (%)	123 (52.3)	60 (56.6)	33,613 (55.7)
Medical history, n (%)			
Severe comorbidities			
Any severe comorbidity	34 (14.5)	27 (25.5)	10,142 (17.0)
Very severe cardiovascular disease	2 (1.3)	4 (5.1)	1021 (2.5)
Severe respiratory disease	5 (3.2)	1 (1.3)	1650 (4.0)
Chronic renal disease	6 (3.8)	2 (2.6)	1122 (2.7)
Chronic liver disease	9 (5.7)	6 (7.6)	1909 (4.7)
Metastatic disease	2 (1.3)	1 (1.3)	1421 (3.5)
Haematological malignancy	3 (1.9)	5 (6.4)	1064 (2.6)
Immunocompromised	16 (10.2)	14 (18.0)	4092 (10.0)
Diabetes mellitus	36 (15.3)	20 (18.9)	9608 (15.9)
Neutropenia	7 (3.7)	2 (2.5)	930 (1.8)
Surgery within up to 7 days prior to admission, n (%)			
Emergency/urgent	91 (38.7)	29 (27.4)	13,127 (21.7)
Scheduled/elective	27 (11.5)	15 (14.2)	14,987 (24.8)
No surgery	117 (49.8)	62 (58.5)	32,230 (53.4)
Acute severity of illness, mean (SD)			
APACHE II Score	19.0 (6.6)	19.6 (7.5)	16.0 (7.0)
ICNARC Physiology Score	23.1 (8.8)	22.4 (9.2)	17.3 (9.3)
Primary reason for admission to the critical care unit, n (%)			
Medical	126 (51.3)	60 (62.5)	32,114 (52.2)
Respiratory	52 (22.4)	24 (23.1)	12,022 (21.0)
Cardiovascular	28 (12.1)	11 (10.6)	5622 (9.8)
Gastrointestinal	15 (6.5)	13 (12.5)	2519 (4.4)
Neurological	6 (2.6)	1 (1.0)	4697 (8.2)
Other	25 (10.8)	11 (10.6)	7254 (12.7)
Surgical	118 (50.2)	44 (41.5)	28,131 (46.6)
Cardiovascular	76 (32.8)	37 (35.6)	13,280 (23.2)
Gastrointestinal	13 (5.6)	5 (4.8)	4201 (7.3)
Other	17 (7.2)	2 (1.9)	7601 (13.3)
Mortality, deaths (%)			
Critical care unit mortality	82 (34.9)	30 (28.3)	10,047 (16.6)
Acute hospital mortality	93 (49.5)	42 (47.7)	13,926 (24.5)
Length of stay (days), median (IQR)			
Critical care unit stay	12 (6, 24)	11 (5, 25)	2 (1, 5)
Unit survivors	12 (6, 25)	12 (6, 26)	2 (1, 5)
Unit non-survivors	12 (7, 23)	10 (3, 25)	2 (1, 6)
Acute hospital stay	33 (15, 58)	40 (20, 73)	13 (6, 27)
Acute hospital survivors	48 (31, 79)	51 (34, 82)	14 (7, 29)
Acute hospital non-survivors	19 (11, 42)	29 (10, 63)	8 (2, 19)

*SD* standard deviation, *IQR* interquartile range

The most common Candida IFD infection site was blood (57 %; Additional file 1: Table S1) corresponding to a rate of candidaemia of 3.3 per 1000 admissions. Of the 359 total admissions with IFD, approximately half had pre-critical care unit IFD (identified from a sample taken pre-admission or on calendar day 1 or 2) with the other half developing IFD during their critical care unit stay (identified from a sample taken from calendar day 3 onwards; Additional file 2: Figure S1). The median day for *Candida* IFD developed during the critical care unit stay was day 7 (IQR 4 to 12). The incidence of *Candida* IFD developed during the critical care unit stay was 3.1 cases per 1000 admissions.

Both *Candida* spp. IFD subgroups received similar organ support and systemic antifungals (Additional file 3: Table S2). In the admissions with *Candida* IFD that received systemic antifungals, 27 % received these before the first sample from which IFD was identified, 47 % within 3 days of the first positive sample and 27 % more than 3 days after the first positive sample.

Characteristics of the patients included in the development and validation samples are reported in the Additional file 4: Table S3. Of 46 potential variables, 19 candidate variables were selected for the admission model and, following backwards elimination, the final model contained seven variables with 11 parameters (c index 0.705; Table 2 and Additional file 5: Table S4). The 24-h model contained seven variables with 10 parameters (c index 0.824) and the end of calendar day 3 model contained five variables with seven parameters (c index 0.835). Validation of the models is reported in Table 3. The end of calendar day 3 model performed the best with a c index of 0.709 in the full validation sample.

**Table 2:** Final risk models at admission, 24 h and end of calendar day 3

	Admission model	24-h model	End of calendar day 3 model
Admission for pre-surgical preparation	5.01 (2.23, 11.26)	–	–
Surgery within 7 days prior to admission ^a^:			
Elective/scheduled – no unexpected complications ^b^	1		
Elective/scheduled – unexpected complications ^b^	2.51 (0.89, 7.06)	–	–
Emergency/urgent	3.61 (2.17, 6.01)	–	–
No surgery	5.59 (2.91, 10.75)	–	–
Surgery within 7 days prior to admission ^c^:			
Elective/scheduled		1	–
Emergency/urgent	–	2.44 (1.28, 4.63)	–
No surgery	–	2.43 (1.12, 5.28)	–
Pancreatitis	4.00 (1.75, 9.15)	3.38 (1.34, 8.56)	3.01 (1.33, 6.85)
Number of central venous catheters:			
None	1	1	1
1	1.49 (1.04, 2.12)	3.87 (1.53, 9.78)	3.65 (1.10, 12.05)
2 or more	4.16 (1.81, 9.60)	13.53 (5.45, 33.60)	14.79 (4.44, 49.26)
Number of drains:			
None	1	1	1
1–3	1.90 (1.23, 2.93)	2.04 (1.41, 2.94)	1.93 (1.26, 2.95)
4 or more	5.12 (1.30, 20.10)	8.05 (2.35, 27.59)	7.61 (2.61, 22.2)
Enteral feeding tube in place	1.52 (1.04, 2.21)	–	–
Lowest SBP (first 24 h) < 90 mmHg	–	1.73 (1.21, 2.46)	–
Highest heart rate (first 24 h) ≥ 100 min^−1^	–	2.35 (1.47, 3.74)	2.20 (1.24, 3.89)
Number of samples positive for fungal colonization ^a^:			
None or 1	1	–	–
2 or more	7.84 (1.54, 39.76)	–	–
Number of samples positive for fungal colonization ^c^:			
None	–	1	1
1 or more	–	6.47 (4.26, 9.84)	8.21 (4.11, 16.39)

Values are odds ratio (95 % confidence interval). *SBP* systolic blood pressure

^a^For admission model

^b^Examples indicative of complications in surgery include: simple surgery with unexpected blood loss (requiring transfusion); unexpected spillage/contamination during surgery; unexpected adhesions making surgery more complex than expected; surgery being far bigger, or lasting far longer than expected

^c^For 24-h/end of calendar day 3 model

**Table 3:** Measures of model performance in the development and validation samples

Measure	Development sample		Validation samples			
	Original	Optimism Adjusted ^a^	Random	Temporal	Geographical	Full
Admission model						
c index	0.705	0.688	0.721	0.650	0.640	0.655
Brier’s score	0.0040	0.0041	0.0026	0.0043	0.0040	0.0038
24-h model						
c index	0.824	0.810	0.840	0.759	0.650	0.732
Brier’s score	0.0038	0.0038	0.0019	0.0042	0.0044	0.0037
End of calendar day 3 model						
c index	0.835	0.825	0.803	0.720	0.661	0.709
Brier’s score	0.0050	0.0050	0.0026	0.0049	0.0048	0.0043

^a^Optimism adjusted using bootstrapping

The comparison of clinical decision rules based on the end of calendar day 3 FIRE Study model with existing clinical decision rules is reported in Table 4. The best performing of the existing rules was rule OZ3. This rule had better sensitivity, specificity, PPV and NPV than rules P1 and P3, while the remaining rules (OZ1, OZ2 and P2) gave higher sensitivity but at the cost of treating 43 to 83 % of admissions. Applying thresholds of >0.5 % (F1) or >1 % (F2) to risk predictions from the FIRE Study model gave similar performance to rule OZ3, with rule F1 giving slightly higher sensitivity but lower specificity and rule F2 giving slightly lower sensitivity but higher specificity (Additional file 6: Figure S2).

**Table 4:** Performance of clinical decision rules at the end of calendar day 3 following admission to the critical care unit in the full validation sample

Rule	Percentage ‘high risk’	Sensitivity (95 % CI)	Specificity (95 % CI)	PPV (95 % CI)	NPV (95 % CI)
FIRE Study					
F1	27.1	54.1 (36.9, 70.5)	73.0 (72.0, 73.9)	0.87 (0.53, 1.34)	99.7 (99.6, 99.8)
F2	15.7	40.5 (24.8, 57.9)	84.5 (83.7, 85.2)	1.13 (0.63, 1.85)	99.7 (99.5, 99.8)
F3	6.4	24.3 (11.8, 41.2)	93.6 (93.1, 94.2)	1.65 (0.76, 3.11)	99.6 (99.5, 99.8)
Ostrosky-Zeichner et al., 2007 [6]					
OZ1	58.5	86.5 (71.2, 95.5)	41.6 (40.5, 42.7)	0.64 (0.44, 0.91)	99.9 (99.7, 100)
OZ2	43.4	81.1 (64.8, 92.0)	56.7 (55.7, 57.8)	0.81 (0.55, 1.16)	99.9 (99.7, 99.9)
OZ3	21.1	51.4 (34.4, 68.1)	79.1 (78.2, 79.9)	1.06 (0.64, 1.65)	99.7 (99.6, 99.8)
Paphitou et al., 2005 [7]					
P1	29.0	43.2 (27.1, 60.5)	71.0 (70.1, 72.0)	0.65 (0.37, 1.05)	99.7 (99.5, 99.8)
P2	83.2	97.3 (85.8, 99.9)	16.8 (16.0, 17.7)	0.51 (0.36, 0.71)	99.9 (99.6, 100)
P3	23.3	43.2 (27.1, 60.5)	76.8 (75.9, 77.7)	0.81 (0.46, 1.31)	99.7 (99.5, 99.8)

*CI* confidence interval, *NPV* negative predictive value, *PPV* positive predictive value

## Discussion

The FIRE Study is the first multicentre study to report specifically on *Candida* IFD in UK critical care units and to develop and validate risk prediction tools to identify critically ill non-neutropenic adults at risk for IFD. *Candida albicans* accounted for two thirds of *Candida* IFD. Blood was the most common infection site, accounting for more than half of all *Candida* IFD. The risk prediction tools were developed at three decision time points: at admission to the critical care unit, at 24 h following admission, and at the end of calendar day 3 following admission. The final model at admission to the critical care unit had fair discrimination (c index ~0.7). When additional information from the first 24 h following admission was added, discrimination improved (c index ~0.8) and this level of discrimination was maintained at the end of calendar day 3. When clinical decision rules were defined based on cut-points of predicted risk at the end of calendar day 3, the performance of these rules was similar to the best performing rule from the literature.

The major strength of the FIRE Study is the huge sample size of admissions to a large number of critical care units across the UK, providing extremely representative and generalisable results. The definition of *Candida* IFD was chosen to be consistent with current international consensus definitions and was carefully validated. However, despite the huge sample size, the low rate of *Candida* IFD observed (although clearly good for patients) made robust statistical modelling challenging. The observed rate of *Candida* IFD was approximately half that anticipated from the literature and consequently the resulting models had a lower number of events per variable than ideal, which may have contributed to the drop in model performance when assessed in the validation samples. Model performance was worst when applied in the geographical validation sample, despite a similar rate of *Candida* IFD, suggesting that particular care should be taken in transferring the models to different geographical settings. A further potential limitation is the reliance on routinely available data – for example, the definition of *Candida* colonisation was based on samples sent for microbiological evaluation in normal clinical practice, and we did not specify any particular screening schedule. This may contribute to variation across critical care units, and therefore to how well the models validated in external data.

The rate of candidaemia in the present study, at 3.3 per 1000 admissions, is similar to other reports from European critical care units [18–20]. A previous hospital-wide surveillance from six sentinel hospitals in the UK identified that 45 % of candidaemia was reported from the critical care unit, corresponding to an incidence of 7.4 per 1000 admissions [21]. A prospective study in 24 French critical care units demonstrated a candidaemia incidence of 6.7 per 1000 admissions [22].

The distribution of *Candida* species in the present study is also similar to that of other critical care units in Western Europe [20]. A retrospective analysis of the EPIC II study, examining *Candida* bloodstream infections in 14,414 patients to 1265 critical care units in 76 countries, demonstrated varying proportions of *Candida albicans* and non-albicans Candida spp. infections [23]. Seventy two percent of the *Candida* infections in Western European units were due to *Candida albicans*, as compared to 66 % in the present study and 79 % in the previous UK sentinel hospital study [21]. Variations in proportions of non-albicans *Candida* spp infections may be due to differential use of fluconazole prophylaxis and subsequent emergence of resistant strains [1]. An analysis of *Candida* isolates reported to the Communicable Disease Surveillance Centre from England and Wales between 1990 and 1999 found that *Candida albicans* was responsible for 60 % of all clinically significant isolates [24]. Annual reporting between 1990 and 1999 showed increasing rates of reported *Candida* species. However, more recent data from the Health Protection Agency have shown a decline in both the total number of *Candida* bloodstream infections and the proportion of these due to *Candida albicans* (51 % in 2010) [25].

A recent systematic review of the literature, conducted as part of the FIRE Study [5], identified only one previous study with the explicit aim of developing a risk model for IFD, the “Candida score” [8]. The Candida score was developed in a cohort of 1699 patients admitted to 73 critical care units in Spain and has subsequently been externally validated among 1107 patients admitted to 36 critical care units in Spain, Argentina and France [26]. There are substantial differences between the rationale and approach of the Candida score and the FIRE Study. The Estudio de Prevalencia de Candidiasis (EPCAN) project, from which the Candida score was developed, recruited only patients staying at least 7 days in the critical care unit, and the Candida score was developed using only those diagnosed with colonisation by *Candida* species at any time during the critical care unit stay. The FIRE Study demonstrated that the risk factors, and the strength of their association with *Candida* IFD, varied between admission to the critical care unit and the end of calendar day 3. A model developed using data from later in the critical care unit stay may therefore not accurately reflect risk earlier in the stay, limiting the usefulness of the Candida score for making early decisions regarding antifungal prophylaxis. Similarly, the comparison of clinical decision rules based on the FIRE Study models with existing rules was limited to the decision time point at the end of calendar day 3 as all previous clinical decision rules have been based solely on data from patients staying at least 3 days [6, 7].

## Conclusion

In summary, incidence of *Candida* IFD in UK critical care units in this study was consistent with reports from other European epidemiological studies, but lower than that suggested by previous hospital-wide surveillance in the UK during the 1990s. Risk modeling using classical statistical methods produced relatively simple risk models, and associated clinical decision rules, that provided acceptable discrimination for identifying patients at ‘high risk’ of *Candida* IFD.

## Additional files

Additional file 1: Table S1. Site of Candida invasive fungal disease (*N* = 359). (DOC 31 kb) Additional file 2: Figure S1. Timing of *Candida* invasive fungal disease relative to critical care unit admission (*N* = 359). (DOC 27 kb) Additional file 3: Table S2. Therapies received, fungal colonisation, mortality and length of stay by invasive fungal disease subgroup. (DOC 61 kb) Additional file 4: Table S3. Description of patients included at each time point in each of the development and validation samples. (DOC 37 kb) Additional file 5: Table S4. Final risk models at admission, 24 h and end of calendar day 3. (DOC 44 kb) Additional file 6: Figure S2. Area under the operating curve for clinical decision rules. (DOC 110 kb)

## Abbreviations

APACHE II: Acute physiology and chronic health evaluation II
EORTC/MSG: European organization for research and treatment of cancer/invasive fungal infections cooperative group and the national institute of allergy and infectious diseases mycoses study group
EPCAN: The estudio de prevalencia de candidiasis
EPIC II: Extended prevalence of infection in the ICU study
FIRE: Fungal infection risk evaluation study
HTA: Health technology assessment
ICNARC: Intensive care national audit & research centre
IFD: Invasive fungal disease
NHS: National health service
NIHR: National institute for health research
