# Supplementary material for: Predicting invasive fungal disease due to Candida species in non-neutropenic, critically ill, adult patients in United Kingdom critical care units
Source: BMC Infect Dis. 2016 Sep 9;16(1):480. doi: 10.1186/s12879-016-1803-9 (PMC5016930; doi:10.1186/s12879-016-1803-9)
Supplement: Additional file 1: Table S1. — Site of Candida invasive fungal disease (N = 359). (DOC 31 kb) [file 12879_2016_1803_MOESM1_ESM.doc]

**Additional file 1**

**Table S1:** Site of Candida invasive fungal disease (N=359)

| **Site of IFD** | **n (%) ^α^** |
| --- | --- |
| Blood | 203 (56.5) |
| Peritoneal fluid | 92 (25.6) |
| Pleural fluid | 36 (10.0) |
| Tissue sample | 7 (1.9) |
| Intravascular catheter | 5 (1.4) |
| Pancreatic fluid | 3 (0.8) |
| Cerebrospinal fluid | 1 (0.3) |
| Pericardial fluid | 1 (0.3) |
| Other sites | 13 (3.6) |

α: Percentages do not add up as two admissions were infected in multiple sites
