# Supplementary material for: Predicting invasive fungal disease due to Candida species in non-neutropenic, critically ill, adult patients in United Kingdom critical care units
Source: BMC Infect Dis. 2016 Sep 9;16(1):480. doi: 10.1186/s12879-016-1803-9 (PMC5016930; doi:10.1186/s12879-016-1803-9)
Supplement: Additional file 2: Figure S1. — Timing of Candida invasive fungal disease relative to critical care unit admission (N = 359). (DOC 27 kb) [file 12879_2016_1803_MOESM2_ESM.doc]

**Additional file 2**

**Figure S1:** Timing of *Candida* invasive fungal disease relative to critical care unit admission (N=359)
