# Supplementary material for: Predicting invasive fungal disease due to Candida species in non-neutropenic, critically ill, adult patients in United Kingdom critical care units
Source: BMC Infect Dis. 2016 Sep 9;16(1):480. doi: 10.1186/s12879-016-1803-9 (PMC5016930; doi:10.1186/s12879-016-1803-9)
Supplement: Additional file 3: Table S2. — Therapies received, fungal colonisation, mortality and length of stay by invasive fungal disease subgroup. (DOC 61 kb) [file 12879_2016_1803_MOESM3_ESM.doc]

**Additional file 3**

**Table S2:** Therapies received, fungal colonisation, mortality and length of stay by invasive fungal disease subgroup

|  | ***Candida albicans* IFD**  (235) | **Non-albicans Candida spp. IFD**  (106) | **No IFD**  (60,362) |
| --- | --- | --- | --- |
| **Therapies received, n (%)** |  |  |  |
| Total parenteral nutrition **^α^** | 44 (18.7) | 18 (17.0) | 2,153 (3.6) |
| Systemic antimicrobial use **^α^** | 222 (94.5) | 104 (98.1) | 51,194 (84.8) |
| Immunospressive use **^α^** | 71 (30.2) | 39 (36.8) | 12,476 (20.7) |
| Central venous catheter **^α^** | 221 (94.0) | 95 (89.6) | 36,387 (60.3) |
| Organ support ^β^ |  |  |  |
| Advanced cardiovascular support | 123 (52.3) | 52 (49.1) | 15,584 (25.8) |
| Advanced respiratory support | 199 (84.7) | 87 (82.1) | 31,380 (52.0) |
| Renal support | 96 (40.9) | 34 (32.1) | 7,123 (11.8) |
| Gastrointestinal support | 201 (85.5) | 84 (79.3) | 23,139 (38.3) |
| Neurological support | 23 (9.8) | 9 (8.5) | 6,612 (11.0) |
| **Outcomes** |  |  |  |
| **Fungal colonisation ^µ^, n (%)** |  |  |  |
| No (including no samples taken) | 17 (7.2) | 4 (3.8) | 54,193 (89.8) |
| Pre-admission | 39 (16.6) | 25 (23.6) | 765 (1.3) |
| Identified in unit | 179 (76.2) | 77 (72.6) | 5,404 (9.0) |
| **Mortality, deaths (%)** |  |  |  |
| Critical care unit mortality | 82 (34.9) | 30 (28.3) | 10,047 (16.6) |
| Acute hospital mortality | 93 (49.5) | 42 (47.7) | 13,926 (24.5) |
| **Length of stay (days), median (IQR)** |  |  |  |
| **Critical care unit stay** | 12 (6, 24) | 11 (5, 25) | 2 (1, 5) |
| Unit survivors | 12 (6, 25) | 12 (6, 26) | 2 (1, 5) |
| Unit non-survivors | 12 (7, 23) | 10 (3, 25) | 2 (1, 6) |
| **Acute hospital stay** | 33 (15, 58) | 40 (20, 73) | 13 (6, 27) |
| Acute hospital survivors | 48 (31, 79) | 51 (34, 82) | 14 (7, 29) |
| Acute hospital non-survivors | 19 (11, 42) | 29 (10, 63) | 8 (2, 19) |

α: During the first 24 hours following admission to the critical care unit

β: At any time during the critical care unit stay

µ: Categorised by time of first report
