# Supplementary material for: Predicting invasive fungal disease due to Candida species in non-neutropenic, critically ill, adult patients in United Kingdom critical care units
Source: BMC Infect Dis. 2016 Sep 9;16(1):480. doi: 10.1186/s12879-016-1803-9 (PMC5016930; doi:10.1186/s12879-016-1803-9)
Supplement: Additional file 4: Table S3. — Description of patients included at each time point in each of the development and validation samples. (DOC 37 kb) [file 12879_2016_1803_MOESM4_ESM.doc]

**Additional file 4**

**Table S3:** Description of patients included at each time point in each of the development and validation samples

|  | **Development sample** | | | **Full validation sample** | | |
| --- | --- | --- | --- | --- | --- | --- |
|  | Admission | 24 hours | day 3 | Admission | 24 hours | day 3 |
| Number of admissions | 35,455 | 26,540 | 16,405 | 18,834 | 13,862 | 8,488 |
| Number of critical care units | 70 | 70 | 70 | 92 | 92 | 92 |
| Age, mean (SD) | 61.4 (17.6) | 61.7 (17.4) | 62.2 (16.9) | 60.4 (17.7) | 60.7 (17.2) | 61.3 (16.5) |
| Male, number (%) | 19,648 (55.4) | 14,893 (56.1) | 9,387 (57.2) | 10,441 (55.4) | 7,736 (55.8) | 4,871 (57.4) |
| Surgery ^a^, number (%) |  |  |  |  |  |  |
| Elective/scheduled | 9,699 (27.4) | 6,494 (24.5) | 3,101 (18.9) | 4,343 (23.1) | 2,757 (19.9) | 1,292 (15.2) |
| Emergency/urgent | 7,651 (21.6) | 5,829 (22.0) | 3,789 (23.1) | 4,107 (21.8) | 3,071 (22.2) | 1,889 (22.3) |
| No surgery | 18,095 (51.1) | 14,211 (53.6) | 9,514 (58.0) | 10,374 (55.1) | 8,026 (57.9) | 5,304 (62.5) |
| Severity scores, mean (SD) |  |  |  |  |  |  |
| ICNARC Physiology Score | 17.0 (9.3) | 17.5 (8.5) | 19.1 (8.2) | 16.7 (9.2) | 17.3 (8.5) | 18.8 (8.1) |
| APACHE II Score | 15.8 (6.9) | 16.3 (6.6) | 17.1 (6.4) | 15.7 (6.9) | 16.1 (6.7) | 16.8 (6.5) |
| *Candida* IFD, number (%) | 144 (0.41) | 104 (0.39) | 85 (0.52) | 72 (0.38) | 52 (0.38) | 37 (0.44) |

*APACHE:* Acute Physiology And Chronic Health Evaluation, *ICNARC:* Intensive Care National Audit & Research Centre, *SD* standard deviation, IFD: invasive fungal disease

^a^ Surgery within up to seven days prior to admission to the critical care unit
