# Supplementary material for: Predicting invasive fungal disease due to Candida species in non-neutropenic, critically ill, adult patients in United Kingdom critical care units
Source: BMC Infect Dis. 2016 Sep 9;16(1):480. doi: 10.1186/s12879-016-1803-9 (PMC5016930; doi:10.1186/s12879-016-1803-9)
Supplement: Additional file 5: Table S4. — Final risk models at admission, 24 h and end of calendar day 3. (DOC 44 kb) [file 12879_2016_1803_MOESM5_ESM.doc]

**Additional file 5**

**Table S4:** Final risk models at admission, 24 hours and end of calendar day 3

|  | **Admission model** | **24-hour model** | **End of calendar day 3 model** |
| --- | --- | --- | --- |
| Admission for pre-surgical preparation | 5.01 (2.23,11.26) | - | - |
| Surgery within 7 days prior to admission ^a^: |  |  |  |
| Elective/scheduled – no unexpected complications ^b^ | 1 |  |  |
| Elective/scheduled – unexpected complications ^b^ | 2.51 (0.89,7.06) | - | - |
| Emergency/urgent | 3.61 (2.17,6.01) | - | - |
| No surgery | 5.59 (2.91, 10.75) | - | - |
| Surgery within 7 days prior to admission ^c^: |  |  |  |
| Elective/scheduled |  | 1 | - |
| Emergency/urgent | - | 2.44 (1.28, 4.63) | - |
| No surgery | - | 2.43 (1.12, 5.28) | - |
| Pancreatitis | 4.00 (1.75, 9.15) | 3.38 (1.34, 8.56) | 3.01 (1.33, 6.85) |
| Number of central venous catheters: |  |  |  |
| None | 1 | 1 | 1 |
| 1 | 1.49 (1.04, 2.12) | 3.87 (1.53, 9.78) | 3.65 (1.10, 12.05) |
| 2 or more | 4.16 (1.81, 9.60) | 13.53 (5.45, 33.60) | 14.79 (4.44, 49.26) |
| Number of drains: |  |  |  |
| None | 1 | 1 | 1 |
| 1-3 | 1.90 (1.23, 2.93) | 2.04 (1.41, 2.94) | 1.93 (1.26, 2.95) |
| 4 or more | 5.12 (1.30, 20.10) | 8.05 (2.35, 27.59) | 7.61 (2.61, 22.2) |
| Enteral feeding tube in place | 1.52 (1.04, 2.21) | - | - |
| Lowest SBP (first 24 hours) < 90 mmHg | - | 1.73 (1.21, 2.46) | - |
| Highest heart rate (first 24 hours) ≥ 100 min^-1^ | - | 2.35 (1.47, 3.74) | 2.20 (1.24, 3.89) |
| Number of samples positive for fungal colonization ^a^: |  |  |  |
| None or 1 | 1 | - | - |
| 2 or more | 7.84 (1.54, 39.76) | - | - |
| Number of samples positive for fungal colonization ^c^: |  |  |  |
| None | - | 1 | 1 |
| 1 or more | - | 6.47 (4.26, 9.84) | 8.21 (4.11, 16.39) |

Values are odds ratio (95% confidence interval). SBP, systolic blood pressure.

^a^ For admission model.

^b^ Examples indicative of complications in surgery include: simple surgery with unexpected blood loss (requiring transfusion); unexpected spillage/contamination during surgery; unexpected adhesions making surgery more complex than expected; surgery being far bigger, or lasting far longer than expected.

^c^ For 24-hour/end of calendar day 3 model
