# Supplementary material for: Predicting invasive fungal disease due to Candida species in non-neutropenic, critically ill, adult patients in United Kingdom critical care units
Source: BMC Infect Dis. 2016 Sep 9;16(1):480. doi: 10.1186/s12879-016-1803-9 (PMC5016930; doi:10.1186/s12879-016-1803-9)
Supplement: Additional file 6: Figure S2. — Area under the operating curve for clinical decision rules. (DOC 110 kb) [file 12879_2016_1803_MOESM6_ESM.doc]

**Additional file 6**

**Figure S2:** Area under the operating curve for clinical decision rules

F1

F2

F3

OZ1

OZ2

OZ3

P1

P2

P3

0

20

40

60

80

100

Sensitivity

0

20

40

60

80

100

1 - Specificity
